# Author Correction: The influence of HLA genotype on the development of metal hypersensitivity following joint replacement

**DOI:** 10.1038/s43856-022-00158-9

**Published:** 2022-07-15

**Authors:** David J. Langton, Rohan M. Bhalekar, Thomas J. Joyce, Stephen P. Rushton, Benjamin J. Wainwright, Matthew E. Nargol, Nish Shyam, Benedicte A. Lie, Moreica B. Pabbruwe, Alan J. Stewart, Susan Waller, Shonali Natu, Renee Ren, Rachelle Hornick, Rebecca Darlay, Edwin P. Su, Antoni V. F. Nargol

**Affiliations:** 1 ExplantLab, The Biosphere, Newcastle Helix, Newcastle-upon-Tyne, England; 2 Newcastle University, Newcastle-upon-Tyne, England; 3 Yale-NUS College, 16 College Avenue West, 138527 Singapore, Singapore; 4 Department of Medical Genetics, University of Oslo and Oslo University Hospital, Oslo, Norway; 5 Royal Perth Hospital, Perth, Australia; 6 School of Medicine, University of St Andrews, St Andrews, Scotland; 7 University Hospital of North Tees, Stockton, England; 8 Hospital for Special Surgery, New York, USA

**Keywords:** Predictive markers, Genetic testing, Bone, Rheumatoid arthritis

Correction to: *Communications Medicine* 10.1038/s43856-022-00137-0, published online 24 June 2022.

In this article the author name Renee Ren was incorrectly written as Renne Ren. The original article has been corrected.

