# Supplementary material for: The influence of HLA genotype on the development of metal hypersensitivity following joint replacement
Source: Commun Med (Lond). 2022 Jun 24;2:73. doi: 10.1038/s43856-022-00137-0 (PMC9232575; doi:10.1038/s43856-022-00137-0)

Supplementary Information

Supplementary Table 1. Extreme phenotype analysis. All class I and class II HLA alleles were typed to six digit resolution. However, only four digit values were used in the comparisons between groups and in the modelling. The full set of allele frequency comparisons is shown in supplementary data table 1.

	**Revised: ALVAL, low wear (27)**	**Revised: Macrophage only (37)**	**Low wear ALVAL vs macrophage only p value**	**Asymptomatic > 10 years (61)**	**ALVAL vs asymptomatic P value**
Male : female	6:21	20:37	**0.010**	45:16	**<0.001**
Median (range) age	65 (42 – 76)	58 (29 – 70)	**0.010**	56 (34 – 75)	**<0.001**
Resurfacings vs THRs	2:25	22:37	**0.003**	61:0	**<0.001**
DQA1*01:01-DQB1*05:01	3/54 (5.5%)	12/74 (15.8%)	0.087	21/122 (17.2%)	**0.026**
DQA1*01:02-DQB1*06:02	9/54 (16.7%)	15/74 (20.2%)	0.772	23/122 (18.4%)	0.891
DQA1*02:01-DQB1*02:02	11/54 (20.3%)	10/74 (13.5%)	0.402	6/122 (4.9%)	**0.015**
DQA1*03:01-DQB1*03:02	3/54 (5.6%)	7/74 (9.5%)	0.404	9/122 (7.4%)	0.651
DQA1*05:01-DQB1*02:01	9/54 (16.7%)	12/74 (16.2%)	1.000	23/122 (12.3%)	0.606
DQA1*05:05-DQB1*03:01	10/54 (18.5%)	3/74 (4.1%)	**0.026**	7/122 (5.7%)	**0.044**

Supplementary Table 2. Cox proportional hazards modelling from phase 1: Centre 1 patients only, N = 161.

Model 1: Survival based on ALVAL severity of mild, moderate or severe						
**Variable**	**Coeff**	**Standard error**	**P value**	**Hazard ratio (HR)**	**HR Lower CI (95%)**	**HR Upper CI (95%)**
Log normalised cobalt concentration	1.575	0.153	<0.001	4.829	3.577	6.518
Age	0.015	0.014	0.308	1.015	0.987	1.044
Rank binding affinity for NTS	-1.062	0.435	0.015	0.346	0.147	0.812
Sex-M	-0.430	0.216	0.047	0.651	0.426	0.994
Type-THR	0.414	0.231	0.074	1.512	0.961	2.380
**Model 2: Survival based on ALVAL severity of moderate or severe**						
Rank binding affinity for NTS	-2.161	0.573	<0.001	0.115	0.038	0.354
Log normalised cobalt concentration	1.598	0.200	< 0.001	4.941	3.341	7.307
Age	0.033	0.019	0.083	1.034	0.996	1.074
Sex-M	-0.370	0.277	0.182	0.691	0.402	1.189
Type-THR	0.455	0.307	0.139	1.576	0.863	2.879

Supplementary Table 3. Clinical details of patients who underwent revision of their prostheses.

ALVAL severity	**Absent**	**Mild**	**Moderate**	**Severe**
Total number of patients	50	50	47	29
Follow up (years)	7 (3 – 15)	6 (2 – 13)	6 (1 – 12)	4 (1 – 12)
Age (range)	57 (29 -79)	57 (25 – 85)	58 (41 – 76)	59 (36 – 74)
% male patients	54% (27:50)	42% (21:39)	43% (20:27)	31% (9:21)
Resurfacings vs THRs	38% (19:31)	54% (27:23)	52% (25:23)	38% (11:29)
BMI	26.7	26.4	24.3	25.5
Median (range) Co (µg/l)	7.2 (0.7 – 271)	7.2 (0.9 – 138.7)	8.0 (1.3 – 178.8)	9.1 (1.8 - 137.5)
Median (range) Cr (µg/l)	6.6 (0.8 – 69.8)	6.8 (0.7 – 108.4)	8.0 (1.1 – 57.9)	7.7 (1.9 – 67.1)
Mean annual volumetric wear rate (mm^3/year)	2.00 (1.0 – 96.0)	2.60 (0.7 – 36.0)	2.78 (0.6 – 36.0)	3.40 (0.8 – 84.0)

Supplementary Table 4. Demographics and clinical details of the training and validation datasets.

	**Data set 1 (training)**	**Data set 2 (test)**
Total number of patients	405	201
Follow up (years)	10 (1 – 17)	10 (1 – 20)
Age (range)	56 (25 – 85)	55 (25 – 81)
% male patients	(65%) 264:141	(66%) 133:68
Resurfacings vs THRs	(78%) 314:91	(77%) 154:47
% patients with bilateral prostheses	26% (105:300)	21% (43:148)
UK/US/Australia	216/173/16	104/86/11
% failed	28% (115)	30% (60)
% mild/moderate/severe ALVAL	20.2% (82/405)	22.3% (45/201)
BMI	26.5	26.8
Median (range) Co (µg/l)	2.0 (0.1 – 271.0)	2.0 (0.1 – 137.5)
Median (range) Cr (µg/l)	2.5 (0.2 – 108.4)	2.4 (0.4 – 58.2)

Supplementary Table 5. This table 1 shows the results of performance evaluation of the presented models on the test set. Taper-dominated wearing THRs were excluded from the test set for the ALVAL pre-operative model to better fit the clinical context in which this model would be exposed to. As the resulting test sets were different for pre-operative and post-operative models, their results are not directly comparable. Similarly high performance was observed across each of the models.

Variable	ALVAL (pre-operative)	ALVAL (post-operative)
**Model**	Gradient boosted survival analysis (regression tree based learner; Cox-PH loss function)	Gradient boosted survival analysis (regression tree based learner; Cox-PH loss function)
**Test data (n)**	Blinded test set excluding taper wear dominated THRs (184)	Blinded test set (201)
**Uno's C-index (95% CI)**	0.862 (0.806 – 0.912)	0.862 (0.809 – 0.908)
**IBS (95% CI)**	0.062 (0.043 – 0.083)	0.066 (0.047 – 0.087)
**Mean AUROC(t) (95% CI)**	0.915 (0.868 – 0.953)	0.915 (0.879 – 0.946)
**ICI at T ≤ t years**		
**t = 2**	0.005	0.011
**t = 3**	0.009	0.016
**t = 4**	0.019	0.023
**t = 5**	0.023	0.019
**t = 6**	0.029	0.016
**t = 7**	0.024	0.013

**t = 8**	0.020	0.023
**t = 9**	0.017	0.051
**t = 10**	0.018	0.031

Supplementary Figure 1. Time dependent AUROC(t) for the pre-operative model from two to ten years after implantation. The shaded area represents the 95% confidence intervals calculated via the Bootstrap method.

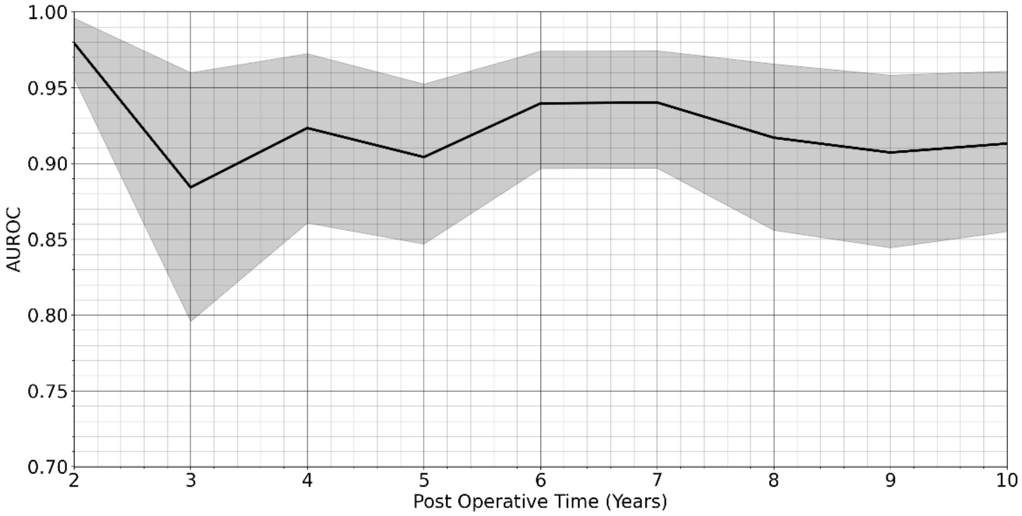

Supplementary Figure 2. Time dependent AUROC(t) for the post-operative model. The shaded area represents the 95% confidence intervals calculated via the Bootstrap method.

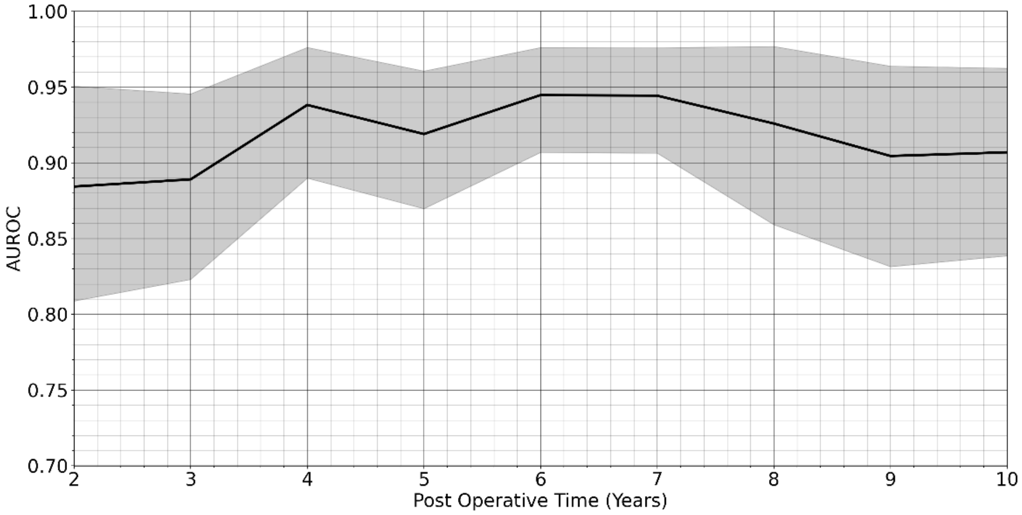

Supplement: Supplementary file 1 — Supplementary Information [file 43856_2022_137_MOESM1_ESM.pdf]
