# Supplementary material for: The influence of HLA genotype on the development of metal hypersensitivity following joint replacement
Source: Commun Med (Lond). 2022 Jun 24;2:73. doi: 10.1038/s43856-022-00137-0 (PMC9232575; doi:10.1038/s43856-022-00137-0)
Supplement: Supplementary file 4 — Description of Additional Supplementary Files [file 43856_2022_137_MOESM4_ESM.pdf]

Description of Additional Supplementary Files

File Name: Supplementary Data 1

Description: Raw genetic data of the extreme phenotype groups are included in Supplementary Data 1

File Name: Supplementary Data 2

Description: Supplementary Data 2 provides the source data for Figures 4, 5 and 6
