# Supplementary material for: The influence of HLA genotype on the development of metal hypersensitivity following joint replacement
Source: Commun Med (Lond). 2022 Jun 24;2:73. doi: 10.1038/s43856-022-00137-0 (PMC9232575; doi:10.1038/s43856-022-00137-0)
Supplement: Supplementary file 6 — Reporting Summary [file 43856_2022_137_MOESM6_ESM.pdf]

nature research

Corresponding author(s): David Langton

Last updated by author(s): 2022/05/27

Reporting Summary

Nature Research wishes to improve the reproducibility of the work that we publish. This form provides structure for consistency and transparency in reporting. For further information on Nature Research policies, see our Editorial Policies and the Editorial Policy Checklist.

Statistics

For all statistical analyses, confirm that the following items are present in the figure legend, table legend, main text, or Methods section.

n/a	Confirmed	
☐	☒	The exact sample size (*n*) for each experimental group/condition, given as a discrete number and unit of measurement
☐	☒	A statement on whether measurements were taken from distinct samples or whether the same sample was measured repeatedly
☐	☒	The statistical test(s) used AND whether they are one- or two-sided<br>*Only common tests should be described solely by name; describe more complex techniques in the Methods section.*
☐	☒	A description of all covariates tested
☐	☒	A description of any assumptions or corrections, such as tests of normality and adjustment for multiple comparisons
☐	☒	A full description of the statistical parameters including central tendency (e.g. means) or other basic estimates (e.g. regression coefficient) AND variation (e.g. standard deviation) or associated estimates of uncertainty (e.g. confidence intervals)
☐	☒	For null hypothesis testing, the test statistic (e.g. *F*, *t*, *r*) with confidence intervals, effect sizes, degrees of freedom and *P* value noted<br>*Give P values as exact values whenever suitable.*
☒	☐	For Bayesian analysis, information on the choice of priors and Markov chain Monte Carlo settings
☒	☐	For hierarchical and complex designs, identification of the appropriate level for tests and full reporting of outcomes
☒	☐	Estimates of effect sizes (e.g. Cohen's *d*, Pearson's *r*), indicating how they were calculated

Our web collection on statistics for biologists contains articles on many of the points above.

Software and code

Policy information about availability of computer code

Data collection	Microsoft Excel
Data analysis	- Python 3.8 - Python packages: o Numpy 1.20.3 o Pandas 1.2.0 o Scipy 1.6.1 o Scikit-learn 0.24.2 o Scikit-survival 0.15.1 o Boruta 0.3<br>Microsoft Excel XLSTAT

For manuscripts utilizing custom algorithms or software that are central to the research but not yet described in published literature, software must be made available to editors and reviewers. We strongly encourage code deposition in a community repository (e.g. GitHub). See the Nature Research guidelines for submitting code & software for further information.

Data

Policy information about availability of data

All manuscripts must include a data availability statement. This statement should provide the following information, where applicable:
- Accession codes, unique identifiers, or web links for publicly available datasets
- A list of figures that have associated raw data
- A description of any restrictions on data availability

Patient consent was not obtained to share the individual genetic results on a public repository. However, further data that support the findings of this study are available from the corresponding author upon reasonable request following ethical approval. Code availability statement: The computer code software algorithm is proprietary and therefore not available to the general reader. The code can be supplied from the corresponding author upon reasonable request for use in non commercial, ethically approved research

Field-specific reporting

Please select the one below that is the best fit for your research. If you are not sure, read the appropriate sections before making your selection.

☒ Life sciences ☐ Behavioural & social sciences ☐ Ecological, evolutionary & environmental sciences

For a reference copy of the document with all sections, see nature.com/documents/nr-reporting-summary-flat.pdf

Life sciences study design

All studies must disclose on these points even when the disclosure is negative.

Sample size	Multiphase study. No previous evidence. Basic power analysis indicated we would need a minimum of 25 patients in the extreme phenotype groups.
Data exclusions	Complex, multi part - largely based on wear or metal ion levels as described in full in methods.
Replication	Tested by developing a model based on 70% of the full data set with the remaining 30% used to test.
Randomization	Not applicable.
Blinding	The lead investigator did not take part in validation of the modelling. The modellers were blinded to the validation data set.

Reporting for specific materials, systems and methods

We require information from authors about some types of materials, experimental systems and methods used in many studies. Here, indicate whether each material, system or method listed is relevant to your study. If you are not sure if a list item applies to your research, read the appropriate section before selecting a response.

Materials & experimental systems

n/a	Involved in the study	
☒	☐	Antibodies
☒	☐	Eukaryotic cell lines
☒	☐	Palaeontology and archaeology
☒	☐	Animals and other organisms
☐	☒	Human research participants
☒	☐	Clinical data
☒	☐	Dual use research of concern

Methods

n/a	Involved in the study	
☒	☐	ChIP-seq
☒	☐	Flow cytometry
☒	☐	MRI-based neuroimaging

Human research participants

Policy information about studies involving human research participants

Population characteristics	Patients who had received metal on metal hip replacements in the preceeding 20 years. Patients were either asymptomatic or their prostheses had failed secondary to adverse reactions to metal debris.
Recruitment	Identified from an existing database. Potential for selection bias however response rates were roughly equal between groups
Ethics oversight	HRA ethics approval IRAS14119 and 227785 study sponsor Newcastle Hospitals Trust LREC County Durham and Tees 2

Note that full information on the approval of the study protocol must also be provided in the manuscript.
